# Overexpression of Allene Oxide Cyclase Improves the Biosynthesis of Artemisinin in *Artemisia annua* L.

**DOI:** 10.1371/journal.pone.0091741

**Published:** 2014-03-18

**Authors:** Xu Lu, Fangyuan Zhang, Qian Shen, Weimin Jiang, Qifang Pan, Zongyou Lv, Tingxiang Yan, Xueqing Fu, Yuliang Wang, Hongmei Qian, Kexuan Tang

**Affiliations:** 1 Plant Biotechnology Research Center, School of Agriculture and Biology, Shanghai Jiao Tong University, Shanghai, China; 2 State Key Laboratory of Natural Medicines, China Pharmaceutical University, Nanjing, China; National Taiwan University, Taiwan

## Abstract

Jasmonates (JAs) are important signaling molecules in plants and play crucial roles in stress responses, secondary metabolites' regulation, plant growth and development. In this study, the promoter of *AaAOC*, which was the key gene of jasmonate biosynthetic pathway, had been cloned. GUS staining showed that *AaAOC* was expressed ubiquitiously in *A. annua*. *AaAOC* gene was overexpressed under control of 35S promoter. RT-Q-PCR showed that the expression levels of *AaAOC* were increased from 1.6- to 5.2-fold in *AaAOC-*overexpression transgenic *A. annua*. The results of GC-MS showed that the content of endogenous jasmonic acid (JA) was 2- to 4.7-fold of the control level in *AaAOC*-overexpression plants. HPLC showed that the contents of artemisinin, dihydroartemisinic acid and artemisinic acid were increased significantly in *AaAOC*-overexpression plants. RT-Q-PCR showed that the expression levels of *FPS* (farnesyl diphosphate synthase), *CYP71AV1* (cytochrome P450 dependent hydroxylase) and *DBR2* (double bond reductase 2) were increased significantly in *AaAOC*-overexpression plants. All data demonstrated that increased endogenous JA could significantly promote the biosynthesis of artemisinin in *AaAOC*-overexpression transgenic *A.annua*.

## Introduction

Jasmonates (JAs) [jasmonic acid (JA) and methyl jasmonate (MeJA)] help to regulate diverse aspects of plant biology that range from stress responses to development [1]. As a signal molecule, JA plays an important role in wound response and pathogenesis [2]–[4]. Additionally, the stress hormone JAs can globally promote the production of multiple primary and secondary metabolites in plants [5]–[7].

**Figure 1:**
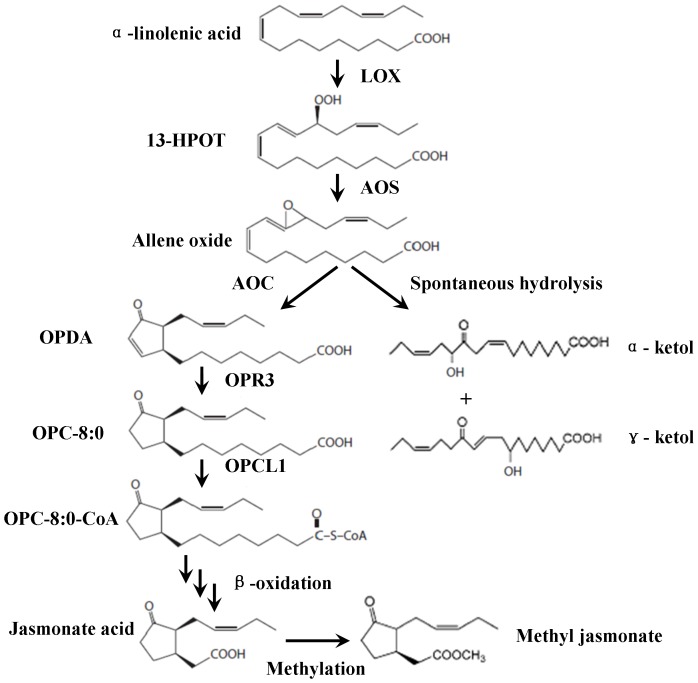
Jasmonate biosynthetic pathway in plants. LOX, 13-lipoxygenase; AOS, allene oxide synthase; AOC, Allene oxide cyclase; OPR3, OPDA reductase 3; OPCL1, OPC-8:0 CoA ligase 1; 13-HPOT, 13-hydroperoxylinoleic acid; OPDA, (9S, 13S)-12-oxo-phytodienoic acid; OPC-8:0, 3-oxo-2(2′-[Z]-pentenyl)cyclopentane-1-octanoic acid; OPC-8:0-CoA, 3-oxo-2-(cis-2′-pentenyl)-cyclopentane-1-octanoyl CoA.

The jasmonate biosynthetic pathway in plants is from an oxylipin pathway (Figure 1) [8]. The α-linolenic acid (18∶3) is converted to 13-hydroperoxylinolenic acid (13-HPOT) by 13-lipoxygenase (LOX), and then allene oxide synthase (AOS) produces allene oxide [1], [9]. The key enzyme Allene oxide cyclase (AOC) catalyses the formation of (9S, 13S)-12-oxo-phytodienoic acid (OPDA) and establishes the stereochemical configuration of naturally occurring JA [10]. Spontaneous hydrolysis of the allene oxide also yields OPDA, although in its racemic form. An OPDA reductase (OPR3) catalyses OPDA to 3-oxo-2(2′-[Z]-pentenyl)cyclopentane-1-octanoic acid (OPC-8:0), which is activated by a carboxyl-CoA ligase encoded by OPCL1 [11]–[14]. Then, after three cycles of β-oxidation, (3R, 7S)-jasmonic acid is produced [15]. MeJA is the ester derivate of JA [16].

As allene oxide cyclase (AOC) is regarded as the most critical step in jasmonate biosynthetic pathway, the gene is preferentially chosen in metabolic engineering of jasmonates. Overexpression of *HnAOC* in tobacco resulted in the overexpression of nicotine biosynthetic pathway genes and higher yield of nicotine, with the maximum of 4.8-fold over control [6]. As well as in *S. miltiorrhiza* hairy roots, overexpression of *SmAOC* significantly enhanced the expression levels of key genes involved in the biosynthetic pathway of diterpenes and phenolic acids and yields of tanshinone IIA, rosmarinic acid (RA) and lithospermic acid B (LAB) [7]. Artemisinin, a functional secondary metabolite of *Artemisia annua*, is currently the best therapeutic against both drug-resistant and cerebral malaria-causing strains of *Plasmodium falciparum* [17]. In this study, we analyzed the results of overexpression *AaAOC* in transgenic *A. Annua* and demonstrated that the increase of endogenous JA resulted in the enhancing of artemisinin biosynthesis.

## Results

**Figure 2:**
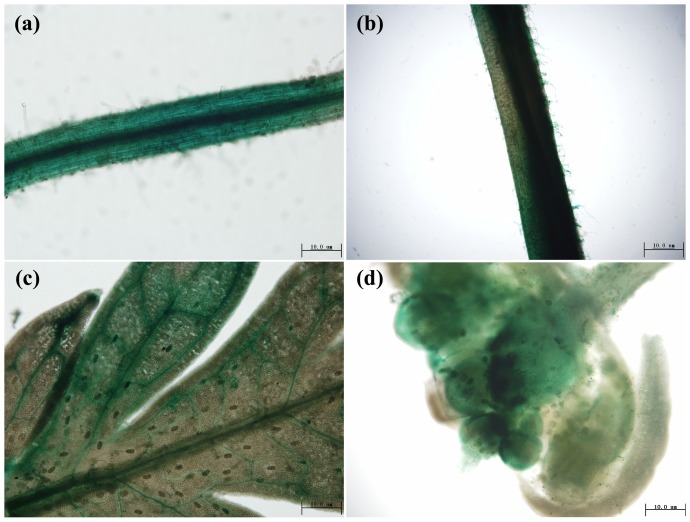
Gus-staining in transgenic *A. annua* transformed using the pAOC-GUS plasmid. (a) Gus-staining of roots in pAOC-GUS transgenic *A. annua*. (b) Gus-staining of stems in pAOC-GUS transgenic *A. annua*. (c) Gus-staining of leaves in pAOC-GUS transgenic *A. annua*. (d) Gus-staining of flower buds in pAOC-GUS transgenic *A. annua*.

To examine the expression pattern of *AaAOC* in detail, a 2276 bp promoter sequence(KC477937) of *AaAOC* was cloned by genomic walking. After generated the *AaAOC* promoter-GUS transgenic plants, the expression pattern of *AaAOC* was investigated by GUS staining. GUS activity was detected in all examined tissues, including roots, stems, leaves and flower buds (Figure 2a, 2b, 2c and 2d). In 1-month-old plants, GUS activity was high in root tips, stems and leaves (Figure 2a, 2b and 2c), along with which can be detected in glandular trichomes and T-shaped trichomes (Figure 2b and 2c). During the flowering period, the GUS staining was observed in flower buds, too (Figure 2d). No GUS staining signals were observed in the negative control plants transformed with pCAMBIA1391Z empty vector (Figure S1 in File S1). All the data showed that *AaAOC* was ubiquitously expressed in *A. annua*.

Independent transformants were selected in kanamycin-containing medium and further confirmed by genomic PCR. Using forward primer of P35S and reverse primer AaAOC-RT-R, 945-bp products were amplified with five transgenic lines AOC-1, AOC-7, AOC-11, AOC13, AOC-17 and the plasmid pCAMBIA2300-35S::*AaAOC*::NOS as template (Figure S2a in File S1). 635-bp products could be amplified from all the transgenic *A. annua* with P35S and NPTII-R as primers (Figure S2b in File S1). The results showed that the existence of *NPTII* gene and exogenous *AaAOC* gene in *AaAOC*-overexpression transgenic plants.

**Figure 3:**
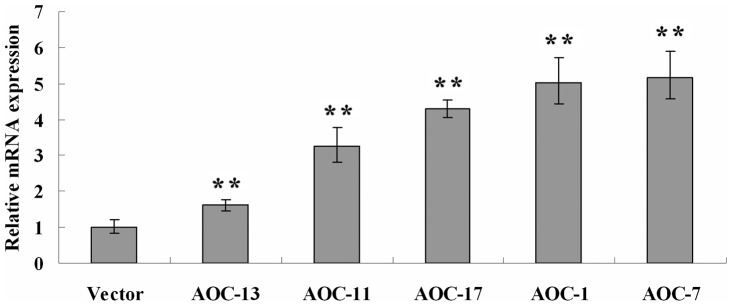
Analysis of *AaAOC*-overexpression transgenic *A. annua* by RT-Q-PCR. The expression analysis of *AaAOC* in transgenic *A. annua* by RT-Q-PCR. Error bars are SE (n = 3). *ACTIN* was used as a control for normalization. Statistical significance was determined by students't-test (**, P<0.01; *, P<0.05). These asterisks are the difference between the empty-vector and *AaAOC*-overexpression transgenic *A. annua*.

The expression levels of *AaAOC* in *AaAOC*-overexpression *A. annua* were analyzed by RT-Q-PCR. The result indicated that expression profile of *AaAOC* gene varied from different transgenic lines. Compared to the control, the expression levels of *AaAOC* were increased from 1.6- to 5.2-fold in *AaAOC*-overexpression transgenic *A. annua* (Figure 3). The statistics analysis showed that the observed differences were statistically significant.

**Figure 4:**
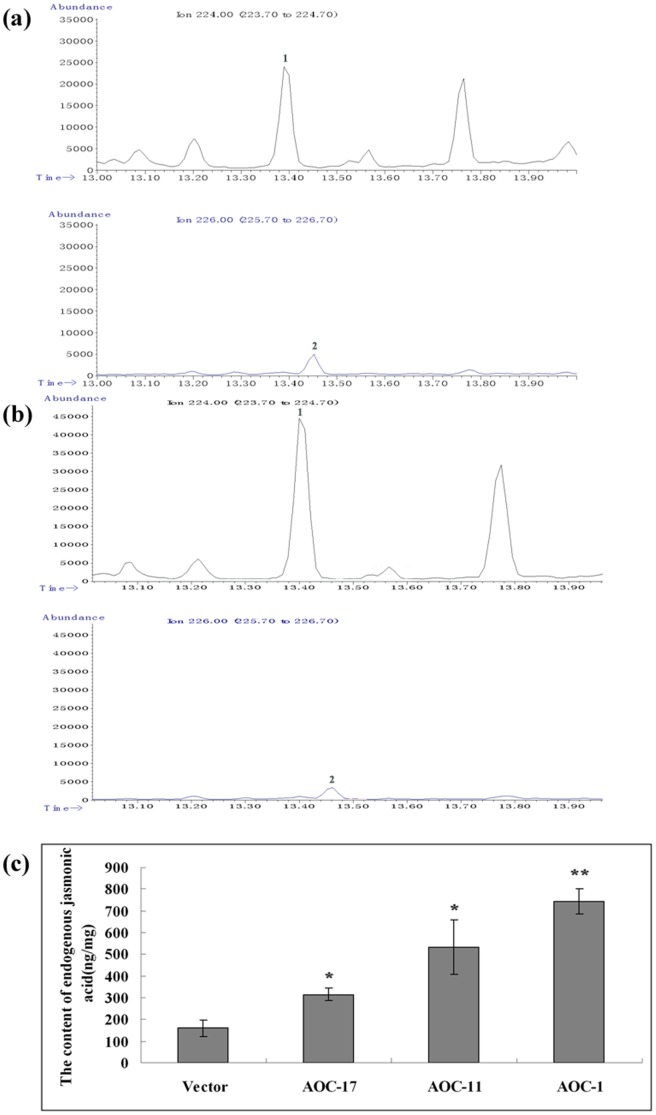
The measurement of endogenous JA by GC-MS. (a) Chromatograms of JA and DHJA in empty-vector transgenic lines. (b) Chromatograms of JA and DHJA in *AaAOC*-overexpression transgenic lines. (c) The content of endogenous JA in empty-vector transgenic plants and *AaAOC*-overexpression transgenic plants. Error bars are SE (n = 3). Statistical significance was determined by students't-test (**, P<0.01; *, P<0.05). These asterisks are the difference between the empty-vector and *AaAOC*-overexpression transgenic *A. annua*.

Three independent transgenic lines were chosen for further analysis. Comparing with the chromatograms and mass spectrometry of JA and DHJA standards, the special peaks of JA and DHJA were found. The retention time of JA was at 13.399 min, while the retention time of DHJA was at 13.456 min (Figure S3a and S3b in File S1). Then, the special peaks of 224 m/z and 226 m/z were extracted and integral, respectively. The ratio of those two peaks were used to count the concentrations of JA. The results showed that the contents of JA were increased 2- to 4.7-fold compared to the control (Figure 4). The statistics analysis showed that the observed differences were statistically significant. All the results demonstrated that the content of endogenous JA increased significantly in *AaAOC*-overexpression transgenic *A. annua*. The growth of *AaAOC*-overexpression lines were barely or only slightly changed versus the control (Figure S4 in File S1).

**Figure 5:**
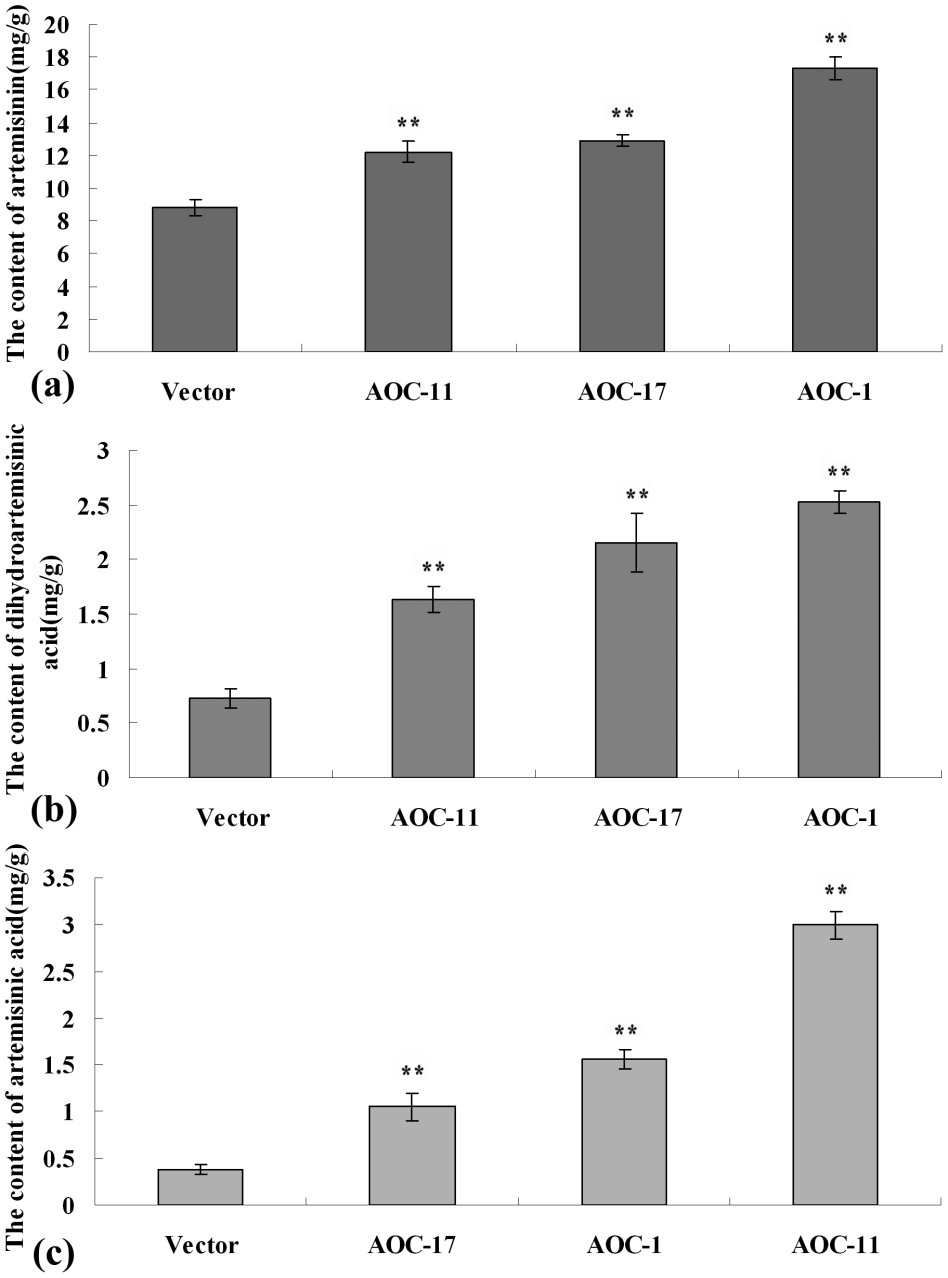
Measurements of artemisinin, dihydroartemisinic acid and artemisinic acid by HPLC in transgenic *A. annua*. (a) The content of artemisinin in empty-vector and three independent *AaAOC*-overexpression transgenic *A. annua*. (b) The content of dihydroartemisinic acid in empty-vector and three independent *AaAOC*-overexpression transgenic *A. annua*. (c) The content of artemisinic acid in empty-vector and three independent *AaAOC*-overexpression transgenic *A. annua*. Error bars are SE (n = 3). Experiments were repeated triplicates.

In view of exogenous JAs can increase the contents of sesquiterpenoids [18], to examine whether increased endogenous JA could enhance the biosynthesis of artemisinin, mature leaves of 5-month-old plants were analyzed by HPLC. Compared to the control, the content of artemisinin was increased by 38–97% in *AaAOC*-overexpression transgenic plants (Figure 5a). Compared to the control, the content of dihydroartemisinic acid was increased by 125–248%, while the content of artemisinic acid was increased by 172–675% (Figure 5b and 5c). The statistics analysis showed that the observed differences were statistically significant.

**Figure 6:**
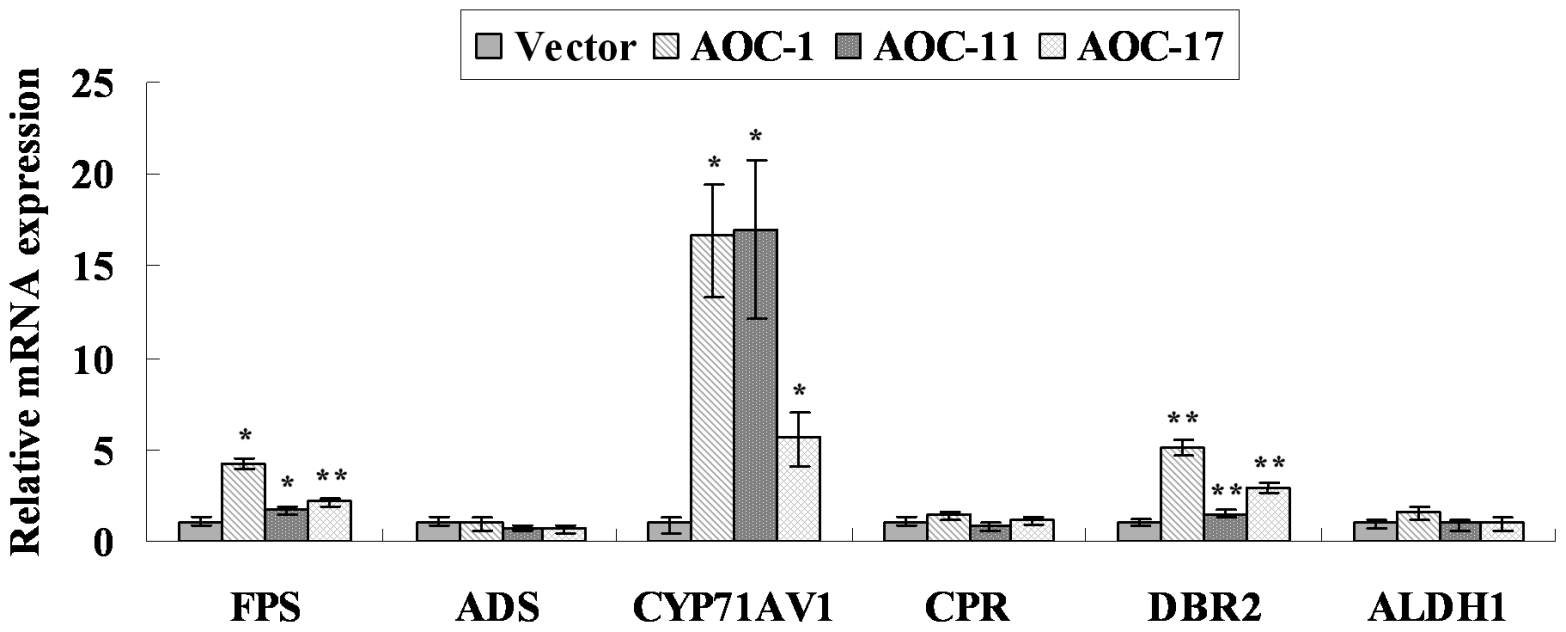
Expression analyses of artemisinin biosynthetic pathway key genes in transgenic *A. annua* by RT-Q-PCR. *FPS*, farnesyl diphosphate synthase gene; *ADS*, amorphadiene synthase gene; *CYP71AV1*, cytochrome P450 dependent hydroxylase gene; *CPR*, cytochrome P450 reductase gene; *DBR2*, double bond reductase 2 gene; *Aldh1*, aldehyde dehydrogenase 1 gene.

The increased accumulation of sesquiterpenoids promoted us to analyse the reasons. Therefore, we analyzed the transcript levels of related genes in artemisinin biosynthesis by RT-Q-PCR (Figure S5 in File S1). The results were shown in figure 6. Briefly, compared to the control, the transcript levels of *FPS* were increased 1.7- to 4.3-fold, while the expression levels of *CYP71AV1* and *DBR2* were increased 5.8- to 17-fold and 1.5- to 5.1-fold, respectively (Figure 6). The statistics analysis showed that the observed differences were statistically significant. The expression levels of *ADS* (amorphadiene synthase), *CPR* (cytochrome P450 reductase) and *ALDH1* (aldehyde dehydrogenase 1) were barely or only slightly changed (Figure 6).

## Discussion

Allene oxide cyclase (AOC) is the key enzyme of jasmonate biosynthetic pathway, which catalyses the formation of OPDA and establishes the stereochemical configuration of naturally occurring JA^10^. Here, our results of GC-MS demonstrated that overexpression of *AaAOC* gene could significantly increase the content of endogenous JA and artemisinin in transgenic *A. annua* plants. Mechanical wounding of Arabidopsis leaves led to an increase in JA-Ile, which is preceded by a large increase in free JA [1], [19], [20]. So, the wounding of *A. annua* plants could increase the content of endogenous JA, while the increased endogenous JA may promote the biosynthetic pathway of artemisinin. The presumption were consistent with the results of Liu *et al.*, which showed that wounding stress can significantly elevate the artemisinin content by increasing the expression levels of some key genes in artemisinin biosynthetic pathway [21]. High level of JA can inhibit the root growth in growth medium [22]. We observed the phenotypes of the control and *AaAOC*-overexpression lines. The growth of *AaAOC*-overexpression lines were only slightly changed versus the control. So, we inferred that the concentrations of JA should reach to a high level which could result in the significant decrease of root growth and plants' growth.

Meas *et al.* showed that exogenous JA treatments promoted the expression levels of artemisinin biosynthetic pathway, which ultimately led to increased artemisinin accumulation in *A. annua* [17]. However, the significance and function of endogenous JA in artemisinin biosynthetic pathway remain unknown in *A. annua*. The results of RT-Q-PCR showed that, compared to the control level, the transcript levels of *FPS*, *CYP71AV1* and *DBR2* were increased significantly in *AaAOC*-overexpression transgenic *A. annua*, while *ADS*, *CPR* and *ALDH1* were barely or only slightly changed. The results of HPLC showed that sesquiterpenoids accumulation significantly increased in *AaAOC*-overexpression transgenic *A. annua*. Interestingly, AOC-1, which has the highest content of endogenous JA, had the highest expression levels of *FPS* and *DBR2* in transgenic *A. annua* plants. The contents of artemisinin and dihydroartemisinic acid in AOC-1 were also the highest in transgenic *A. annua* plants. From the above results, we concluded that the increased endogenous JA significantly promoted the expression levels of some key genes in aretmisinin biosynthetic pathway and resulted in the increase of sesquiterpenoids accumulation in *AaAOC*-overexpression transgenic *A. annua*. All above results demonstrated that the increased endogenous JA could positively regulate the biosynthesis of artemisinin in *AaAOC*-overexpression transgenic *A. annua*. So, endogenous JA also played an important role in the biosynthesis of artemisinin in *A. annua*.

In this study, 2276 bp *AaAOC*-promoter sequence was cloned by genomic walking. GUS staining showed that *AaAOC* was expressed ubiquitiously in *A. annua* plants. In *AaAOC*-overexpression transgenic *A.annua*, the content of endogenous JA was 2- to 4.7-fold of the control. The result of HPLC showed that sesquiterpenoids accumulation was increased significantly in *AaAOC*-overexpression transgenic *A.annua*. RT-Q-PCR showed that the expression levels of *FPS*, *CYP71AV1* and *DBR2* were increased significantly in *AaAOC*-overexpression transgenic *A. annua*. In a word, overexpression of *AaAOC* gene significantly increased the content of endogenous JA in transgenic *A. annua*. And the increased endogenous JA promoted the expression levels of some key genes in artemisinin biosynthetic pathway, which resulted in the increase of artemisinin biosynthesis in *AaAOC*-overexpression transgenic *A. annua* plants.

## Materials and Methods

### Materials

The seeds of *A. annua* were obtained from the School of Life Sciences, Southwest University in Chongqing, P.R. China. Leaves from *A. annua* plants were collected for RNA extraction using plant RNA isolation reagent (Tiangen Biotech, Beijing) following the manufacturer's instructions.

### Clone of *AaAOC* promoter and β-glucuronidase (GUS) staining in transgenic *A. annua*

The upstream region of *AaAOC* was amplified from genomic DNA using the Genome Walker Kit (Clontech, Canada). The *AaAOC*-specific primers (AaAOC-sp1, AaAOC-sp2), Adaptor Primer 1 and Adaptor Primer 2 were used following the manufacturer's recommended procedures. The final reaction products were electrophoresed in 1% agarose gel, and a 2276 bp fragment was eluted from the gel and cloned into the pMD18-T simple vector. The insert DNA was sequenced by Shenzhen Genomics Institute.

To generate the *AaAOC* promoter-GUS construct, the 5′-flanking DNA of the *AaAOC* coding region was amplified with *AaAOC-PF* and *AaAOC-PR*. The 2.2 kb of PCR fragment was cloned into the pCAMBIA1391Z vector. The construct was transformed into *A. annua* plants as described previously [23]. Histochemical staining for GUS activity in transgenic plants was performed as the protocol described previously [24]. Plants transformed with pCAMBIA1391Z were used as a parallel negative control.

### *AaAOC*-overexpression in *A. annua*

AaAOCF and AaAOCR were designed to amplify the ORF of *AaAOC* with *A. annua* cDNA as template. The pMD18-T-AaAOC vector was digested by *Bam*HI and *Sac*I. The full-length ORF of *AaAOC* was cloned into the *Bam*HI and *Sac*I sites of the pCAMBIA2300+ vector under the 35S promoter to generate pCAMBIA2300-35S::*AaAOC*::NOS. The construct was then transferred into *A. tumefaciens* strain EHA105 by the conventional freezing and melting method, and the resulted strains were used in the transformation of *A. annua*. The transformation of *A. annua* was performed following the protocol of Zhang *et al* [23]. All the transgenic plants were grown in the green house for 5 months.

### PCR analysis in transgenic *A. annua*

The genomic DNA for PCR detective was isolated from fresh leaves by using the modified CTAB (cetylmethylammonium bromide) method [25]. The introduced *AOC* gene and *NPTII* gene were respectively detected by primers P35S, AaAOC-RT-R and NPTII-R. PCR reaction was carried out in a 20 µl reaction volume. The thermal cycling conditions was 94°C for 4 min, followed by 35 cycles of amplification (94°C for 30 s, 56°C for 30 s and 72°C for 1 min10 s) and 72°C for additional 10 min.

### Real-time Quantitative PCR analysis (RT-Q-PCR)

The expression levels of *AaAOC* and key genes of artemisinin biosynthetic pathway in *AaAOC*-overexpression *A. annua* were analysed by RT-Q-PCR method. All RNA samples were digested with DNaseI prior to use. Aliquots of 0.4 µg total RNA were employed in the reverse transcriptase reaction using random hexamer primers for the synthesis of first strand cDNA. The amplification reactions of RT-Q-PCR were performed on an iCycler iQTM Real-Time PCR Machines (Bio-Rad, Watford, UK), and the SYBR ExScript RT-PCR kit (Takara, Shiga, Japan) protocol to confirm changes of gene expression. Procedures of RT-Q-PCR were performed as previously described [26]. The *actin* gene was used as the constitutive control gene. All the primers used in RT-Q-PCR were listed in Table S1 in File S1.

### Extraction and qualitative analysis of endogenous JA by GC-MS

The extract procedure was done as described previously with some modifications [27], [28]. Leaves of transgenic and control plants were homogenised in liquid N_2_, then all the powder of samples were frozen dried; 50 mg of dry samples mixed with 1 ml of 2-propanol/water/concentrated HCL (2∶1∶0.002 v/v/v) and added 1000 ng of DHJA as the internal standard, then vortex for 1 min. After sonicate for 30 min, 1 ml of methylene chloride were added to the mixtures and vortex for 1 min; After centrifugation at 3500 rpm for 15 min at 5°C, the bottom methylene chloride/2-propanol layer was collected in new glass tubes. 2 µl of 2M trimethylsilyldiazomethane in hexane was added to the mixture. Cap the vials and allow them to stay at RT for 30 min. 2 µl of 2M acetic acid in hexane was added to the mixture. Dry under gaseous N_2_. Then, 200 µl of methylene chloride was added to reconstituted, then centrifugation at 10000 rpm for 3 min. The supernatant were collected to analyse by GC-MS.

The extracts were analysed using GC-MS (7890A GC/5975C MS, Agilent, USA). The column was DB-5MS (30 m×0.25 mm×0.25 µm). The program was 80°C (2 min hold), from 80°C to 200°C at 10°C/min, from 200°C to 300°C (15 min hold) at 20°C/min, with a splitless injector and electronic pressure control. The MS conditions were EI+ of 70 eV, full scan. The scan range was 33–500 m/z. The compounds were identificated by searching the NIST2011 library and the retention index (minutes) of JA and DHJA standards were obtained.

### Metabolite analysis by High-performance liquid hromatography (HPLC)

Pooled leaves from 10^th^, 15^th^, 20^th^ branches (counted from the apical meristem) of five month-old plants were harvested as samples. The leaves were dried at 45°C and grounded. The dried-leaf powder (0.1 g/sample) was extracted with methanol (2 ml) using a Shanghai Zhishun Instrument Co. Ltd model JYD-650 ultrasonic processor (two bursts of 30 min each). Then, the samples were centrifuged for 10 min at 4000 rpm to remove the suspended particles. The final supernatant was filtered through a 0.25-µm-pore-size filter.

Artemisinin, dihydroartemisinic acid and artemisinic acid were measured using a Waters Alliance 2695 HPLC system. The methods of measure these compounds were described as Ferreira and Gonzalez [29]. The results were analysed using Empower (Waters' chromatography data software).

### Statistical analyses

All the experiments including GUS staining, PCR, real-time quantitative PCR analysis, HPLC analysis and measurement of endogenous JA by GC-MS were repeated three times. Results of metabolites content and real-time quantitative PCR analysis are presented as mean values ± S.D. The error bars are due to biological triplicates. Statistical significance was determined by students't-test (**, P<0.01; *, P<0.05).

## Supporting Information

File S1 Contains the following files: **Figure S1**. Gus-staining of transgenic *A. annua* using the pCAMBIA1391Z empty vector. **Figure S2**. Analysis of *AaAOC*-overexpression transgenic *A.annua* by PCR. (a) PCR analysis using 35S forward primer and AaAOC reverse primer in AaAOC-overexpression transgenic *A. annua*. (b) PCR analysis using 35S forward primer and reverse primer of NPTII gene in AaAOC-overexpression transgenic A. annua. **Figure S3**. Chromatograms and mass spectrometry of JA and DHJA standards. (a) Chromatograms and mass spectrometry of JA. Peak1 is JA and the retention time is 13.399 min. (b) Chromatograms and mass spectrometry of DHJA. Peak2 is DHJA and the retention time is 13.456 min. **Figure S4**. The phenotypes of the transgenic *A.annua* and empty vector line. **Figure S5**. The synthetic pathway of artemisinin in *A.annua*. FPS, farnesyl diphosphate synthase; ADS, amorphadiene synthase; CYP, cytochrome P450 dependent hydroxylase (CYP71AV1); CPR, cytochrome P450 reductase; DBR2, double bond reductase 2; Aldh1, aldehyde dehydrogenase 1. **Table S1**. Primers used in this investigation.
(DOC)
