# Supplementary material for: Overexpression of Allene Oxide Cyclase Improves the Biosynthesis of Artemisinin in Artemisia annua L
Source: PLoS One. 2014 Mar 18;9(3):e91741. doi: 10.1371/journal.pone.0091741 (PMC3958394; doi:10.1371/journal.pone.0091741)
Supplement: File S1 — Contains the following files: Figure S1. Gus-staining of transgenic A. annua using the pCAMBIA1391Z empty vector. Figure S2. Analysis of AaAOC-overexpression transgenic A.annua by PCR. (a) PCR analysis using 35S forward primer and AaAOC reverse primer in AaAOC-overexpression transgenic A. annua. (b) PCR analysis using 35S forward primer and reverse primer of NPTII gene in AaAOC-overexpression transgenic A. annua. Figure S3. Chromatograms and mass spectrometry of JA and DHJA standards. (a) Chromatograms and mass spectrometry of JA. Peak1 is JA and the retention time is 13.399 min. (b) Chromatograms and mass spectrometry of DHJA. Peak2 is DHJA and the retention time is 13.456 min. Figure S4. The phenotypes of the transgenic A.annua and empty vector line. Figure S5. The synthetic pathway of artemisinin in A.annua. FPS, farnesyl diphosphate synthase; ADS, amorphadiene synthase; CYP, cytochrome P450 dependent hydroxylase (CYP71AV1); CPR, cytochrome P450 reductase; DBR2, double bond reductase 2; Aldh1, aldehyde dehydrogenase 1. Table S1. Primers used in this investigation. (DOC) [file pone.0091741.s001.doc]

**Supplement information**


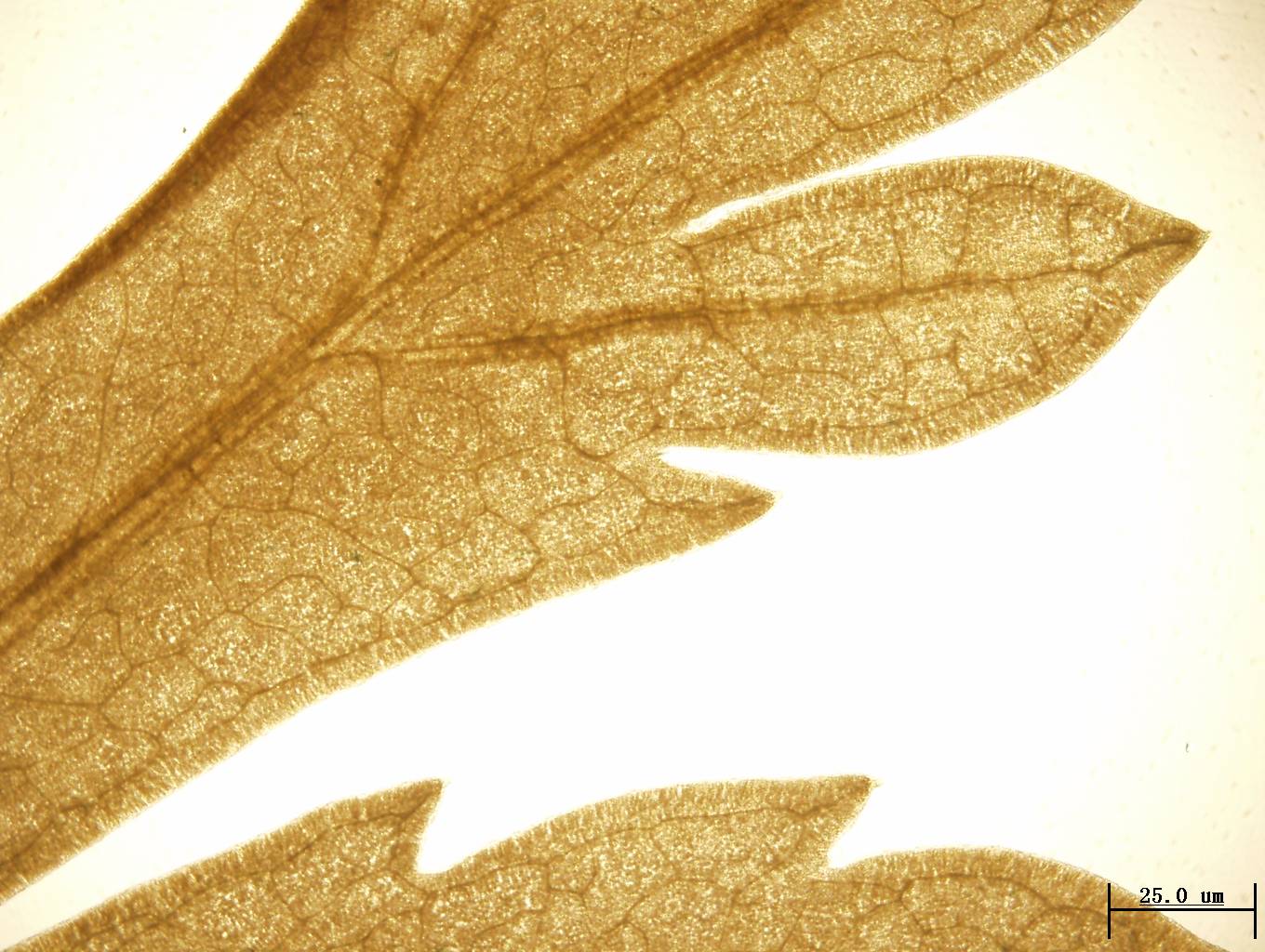


Fig.S1


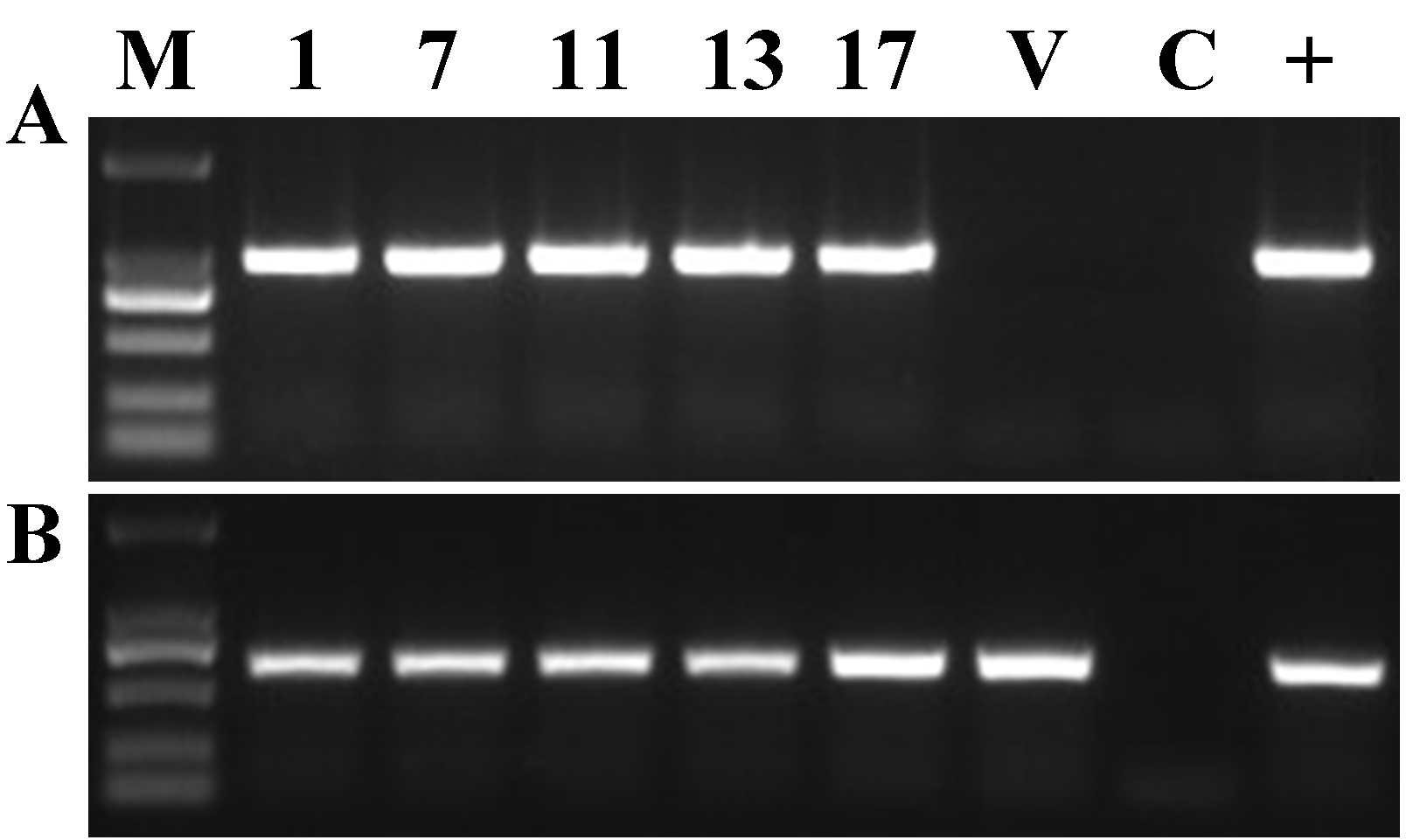


Fig.S2


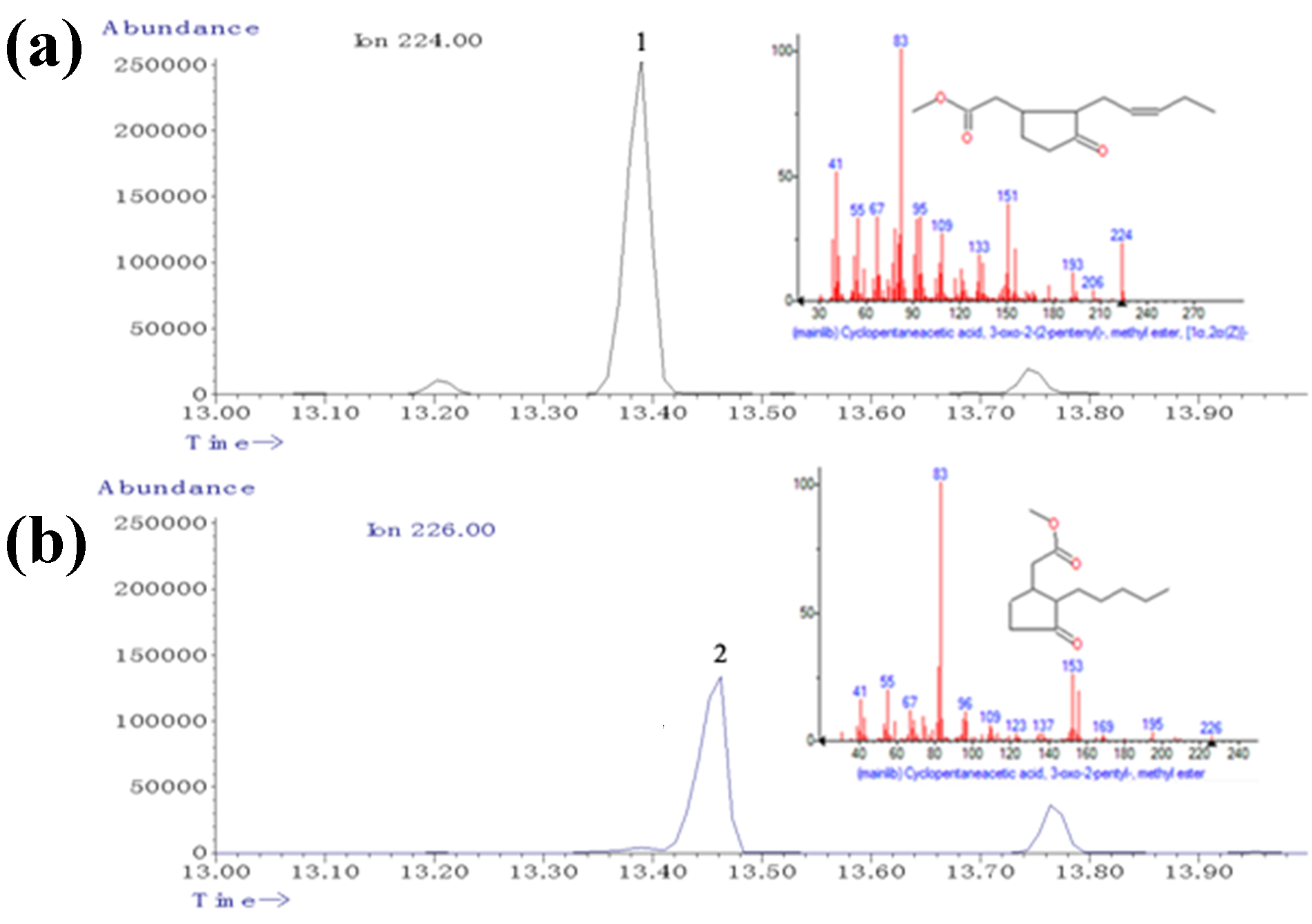


Fig.S3


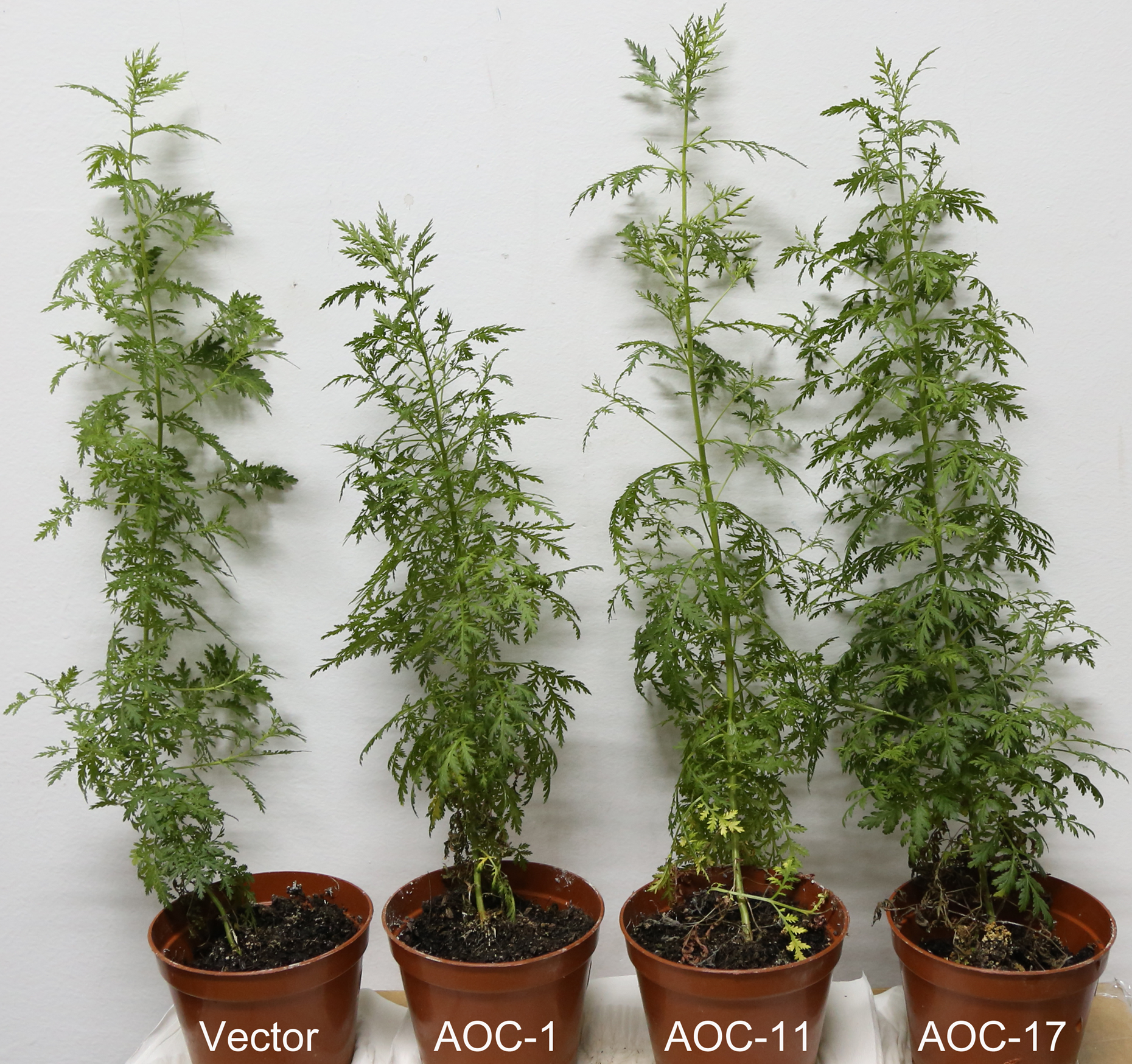


Fig.S4


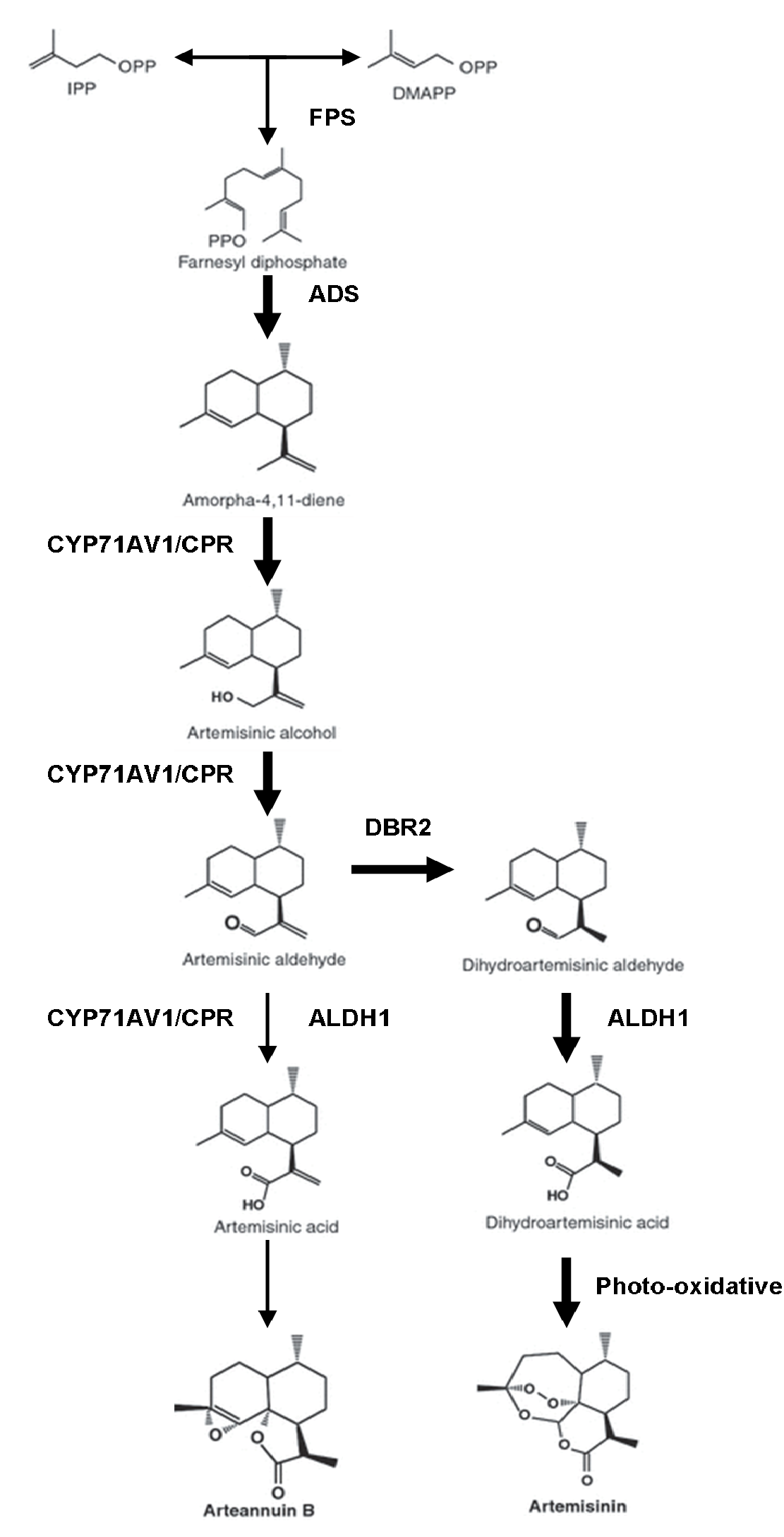


Fig.S5

**Table S1** Primers used in this investigation.

| *Primers Purpose Primer Sequence(5’-3’)* |
| --- |
| AaAOC-sp1 Promoter clone TAAGCCCAAGCCCATAAGAAAAGGTT  AaAOC-sp1 Promoter clone AGCTGTGTTTGACGTGAAGCTAATCG  Adaptor Prime1 Promoter clone GTAATACGACTCACTATAGGGC  Adaptor Prime2 Promoter clone ACTATAGGGCACGCGTGGT  AaAOC-PF Vector contruction CCCTGCAGTCTCACGGTCAACATGGGAG  AaAOC-PR Vector contruction  CCGAATTCAGTTGTTGTTCAAGATTTGTTTTAACG  AaAOCF  Vector contruction CCGGATCCATGGCAGCTGCTGCTTCA  AaAOCR Vector contruction CCGAGCTCTTAATCACTAAAGTTAGGACCA GTGG  AaAOC*-*RT-F Q-PCR GTCCTGCTTACCTACGGTTGGG  AaAOC*-*RT-R Q-PCR, PCR TTCTGTCGTGATGTATGCGCCT  FPS-RT-F Q-PCR TCATTGTCTATTCACCGCCG  FPS-RT-R Q-PCR CACCGCTTGGACTGCTTTGCT  ADS-RT-F Q-PCR AATGGGCAAATGAGGGACAC  ADS-RT-R Q-PCR TTTCAAGGCTCGATGAACTATG  CYP71AV1-RT-F Q-PCR CACCCTCCACTACCCTTG  CYP71AV1-RT-R Q-PCR GACACATCCTTCTCCCAGC  CPR-RT-F Q-PCR AGCCTCTTTGCCACCTCCT  CPR-RT-R Q-PCR GAACAGACTCCCTTGTGAACG  DBR2-RT-F Q-PCR CTTGGGTTACAAGCTGTG GCTCAAG  DBR2-RT-R Q-PCR ATATAATCAAAACTAGAGGAGTGACC  ALDH1-RT-F Q-PCR CA GTTTCTGACCCAAATCCAGGTTGA  ALDH1-RT-R Q-PCR TCGGAGTAGTTGGTCACAT  Actin-F Q-PCR CCAGGCTGTTCAGTCTCTGTAT  Actin-R Q-PCR CGCTCGGTAAGGATCTTCATCA  P35S PCR TTCGTCAACATGGTGGAGCA  NPTII-R PCR CCCTGATGCTCTTCGTCCA |
